# Role of Antidiarrhoeal Drugs as Adjunctive Therapies for Acute Diarrhoea in Children

**DOI:** 10.1155/2013/612403

**Published:** 2013-03-03

**Authors:** Christophe Faure

**Affiliations:** Division of Gastroenterology, Department of Pediatrics, CHU Sainte-Justine, Montreal, QC, Canada H3T 1C5

## Abstract

Acute diarrhoea is a leading cause of child mortality in developing countries. Principal pathogens include *Escherichia coli*, rotaviruses, and noroviruses. 90% of diarrhoeal deaths are attributable to inadequate sanitation. Acute diarrhoea is the second leading cause of overall childhood mortality and accounts for 18% of deaths among children under five. In 2004 an estimated 1.5 million children died from diarrhoea, with 80% of deaths occurring before the age of two. Treatment goals are to prevent dehydration and nutritional damage and to reduce duration and severity of diarrhoeal episodes. The recommended therapeutic regimen is to provide oral rehydration solutions (ORS) and to continue feeding. Although ORS effectively mitigates dehydration, it has no effect on the duration, severity, or frequency of diarrhoeal episodes. Adjuvant therapy with micronutrients, probiotics, or antidiarrhoeal agents may thus be useful. The WHO recommends the use of zinc tablets in association with ORS. The ESPGHAN/ESPID treatment guidelines consider the use of racecadotril, diosmectite, or probiotics as possible adjunctive therapy to ORS. Only racecadotril and diosmectite reduce stool output, but no treatment has yet been shown to reduce hospitalisation rate or mortality. Appropriate management with validated treatments may help reduce the health and economic burden of acute diarrhoea in children worldwide.

## 1. Introduction

Diarrhoeal disease is a major public health concern for both developed and developing countries. Acute diarrhoea is a leading cause of child mortality in developing countries, accounting for 1.5–2 million deaths in children under five years [1]. In consequence, the economic impact of the disease and its treatment are of considerable importance. The aim of the present paper is to provide an update on the aetiology, epidemiology, and treatment of acute diarrhoea in children.

## 2. Definition

Acute diarrhoea is defined as the production of three or more watery stools a day for less than 14 days. In nonsevere acute diarrhoea of gastroenteritic origin, these stools do not contain visible amounts of blood or mucus. If this occurs, then the appropriate diagnosis is dysentery, which requires specific management. The World Health Organization (WHO) emphasises the importance of parental insight in deciding whether children have diarrhoea or not, and in the first few months of life, a conspicuous change in stool consistency rather than stool frequency must be taken into account [2].

## 3. Aetiology of Acute Diarrhoea in Children

Acute infectious diarrhoea results from various viral, bacterial, and parasitic infections and is most frequently of infectious origin. Nonetheless, in about 40% of the cases, no causative agent can be detected [3]. The relative contribution of the different pathogens may vary depending on the specific geographical location and on the season, with acute diarrhoea being predominantly of viral origin in winter and of bacterial origin in summer. Bacterial pathogens are relatively more important in developing countries and viral pathogens relatively more important in developed countries. The principal bacterial pathogen responsible for infectious diarrhoea in children is *Escherichia coli* [1]. With respect to viral pathogens, although rotaviruses were long considered the principal pathogens responsible [1], noroviruses are now seen to be an important emerging viral pathogen and are thought to be the leading cause of nonbacterial gastroenteritis worldwide and a significant cause of mortality in children [4–7].

Rotavirus infection accounts from 20% to 60% of all diarrhoeal episodes in developing and developed countries [8] and is the major cause of acute diarrhoea in young children under five years of age [9]. Although rotavirus infections are usually mild, they may lead to more pronounced watery stool loss, which may sometimes lead to severe dehydration [8, 10–12]. The WHO has estimated that rotavirus infection was responsible for 453,000 deaths in children under 5 years in 2008, accounting for 37% of diarrhoea-related deaths [13].

Noroviruses are highly infectious, and it has been estimated that eighty percent of nonbacterial epidemics of gastroenteritis can be attributed to noroviruses [14, 15]. Other viral pathogens include other caliciviruses such as sapoviruses, adenoviruses and astroviruses [1]. The other main bacterial agents responsible for diarrhoea include *Campylobacter jejuni*; several *Shigella* species; and various *Salmonella* strains including *S. Typhi* and *S. Paratyphi*, the agents of typhoid fever, and *Vibrio cholerae*, the agent of cholera [1, 16]. Of protozoal pathogens, *Entamoeba* species are an important cause of dysentery and may be difficult to eradicate.

These different pathogens may target different parts of the gastrointestinal tract, which may influence the symptomatic manifestations of the disease [17]. For example, *E. coli* and rotaviruses principally infect the small intestine and cause voluminous watery diarrhoea associated with abdominal cramping, bloating, gas, and weight loss. *Shigella* and adenoviruses, on the other hand, principally infect the colon and produce a lower volume diarrhoea often associated with fever and abdominal pain. Infections of the large intestine may frequently lead to the appearance of blood or mucus in the stools (dysentery) [17].

## 4. Disease Severity

The most dangerous symptom of infectious diarrhoea is dehydration, which is the direct cause of many diarrhoeal deaths, principally in infants and young children. The extent of dehydration occurring during a diarrhoeal episode will be inversely related to the total body fluid volume, which is lowest in children. As such, it is very young children, especially in their first two years of life, who are particularly at risk from acute diarrhoea [18]. In developing countries, children are also more likely to be malnourished, which aggravates the risk associated with acute diarrhoeal infections. Severe malnutrition, and in particular, kwashiorkor, may also be the indirect cause of diarrhoea in children. In addition, diarrhoea, especially if it persists over several days, can be a cause of malnutrition in young children due to impaired absorption of amino acids and sugars.

During diarrhoea there is an increased loss of water and electrolytes (sodium, chloride, potassium, and bicarbonate) in the liquid stools. Water and electrolytes are also lost through vomit, sweat, urines and breathing. Dehydration occurs when these losses are not replaced adequately and a deficit of water and electrolytes develops [19]. From a pathophysiological perspective, dehydration arises from an interaction between infectious agents and the intestinal epithelium as well as with underlying cells present in the *lamina propria* [20–23]. Since different microbial pathogens may cause diarrhoea, dehydration in diarrhoea may arise from a variety of mechanisms depending on the specific interaction of the causative agent with intestinal epithelium. Whatever the initial cause, dehydration results from an imbalance between the absorptive and secretory functions of the intestine. On a cellular level, this is characterised by an inhibition of villous absorption and enhanced crypt cell secretion and, as a result, marked loss of water and electrolytes.

## 5. Epidemiology

**Figure 1:**
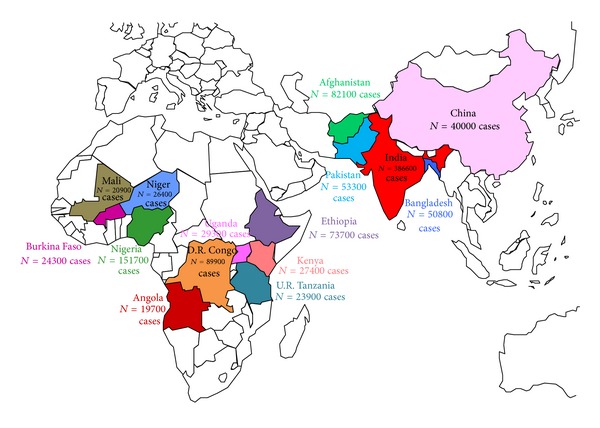
Countries with highest numbers of infantile deaths due to acute diarrhoea. Adapted from [27].

Diarrhoeal diseases are a leading cause of childhood morbidity and mortality in developing countries and an important cause of malnutrition. All forms of childhood diarrhoea are potentially life threatening, and childhood gastroenteritis is the leading cause of mortality of children with 18% of cause-specific deaths among children under five years of age in years 2000–03. In 2003 an estimated 1.87 million children below five years of age died from diarrhoea. Both the incidence and mortality of diarrhoeal diseases are greatest among children younger than one year of age, declining thereafter incrementally [1], with eight out of ten of these deaths occuring in the first two years of life. According to a recent WHO/UNICEF report [24], 15 countries account for almost 75% of all deaths from diarrhoea among children under five years of age each year (Figure 1), and more than 80% of child deaths due to diarrhoea occur in Africa and South Asia. The incidence of acute gastroenteritis in children is especially frequent in areas without access to clean water [25], and it is estimated that about 88% of diarrhoeal deaths worldwide are attributable to unsafe water, inadequate sanitation, and poor hygiene [26].

While efforts to control childhood diarrhoea have resulted in a substantial decline in child deaths over the past three decades, from an estimated five million deaths to around 1.5 million children in 2004 [27] diarrhoea remains the second leading cause of overall childhood mortality after pneumonia [27], and one of the major causes of disability-adjusted life-years (4.7%) [26]. The persistently high rates of morbidity associated with diarrhoea are still of concern, because early childhood diarrhoea may have long-term effects on growth and cognitive function [28]. In developing countries, more than one billion diarrhoea episodes occur every year in children under five years of age (a median of 3.2 episodes of diarrhoea per child-year) in the poorest areas [19, 29].

Rotavirus vaccines were introduced in 2006 and in 2009 their use was recommended by the WHO in all countries. Since then, substantial declines in morbidity and mortality attributed to rotavirus and in all-cause diarrhoea have been seen in countries where vaccination is provided [13]. The reduction in the number of severe cases of all-cause diarrhoea provided by vaccination appears to be more substantial in low-mortality countries [30]. For instance, in the USA, the rate of hospitalisations due to acute diarrhoea during the 2008 rotavirus season was 55.5 per 10,000 children under five years corresponding to a decrease of one-half to two-thirds, compared to the rates observed during the 2000–2006 period prior to vaccination [31, 32]. In El Salvador, hospitalisations among children under five years have decreased by 69%–81% as compared with the prevaccination period [33]. However, in other countries, the impact of vaccination on hospitalisation rates have been reported to be lower; for example, in Mexico, the reduction in hospitalisation was 11%–40% [34, 35]. One systematic review has evaluated the decrease in incidence of cases of severe diarrhoea after vaccination with one of the two available vaccines [30]. Thus, for monovalent rotavirus vaccine, incidence was decreased by 35%–40% in low-mortality countries and by 15%–to 30% in high-mortality countries. After vaccination with pentavalent rotavirus vaccine, incidence was reduced by 73%–96% in low-mortality countries and by 15% in high-mortality countries. However, even though vaccination may have a more modest effect in high-mortality countries, the incidence of diarrhoea is much higher in these countries, and for this reason the absolute number of episodes prevented is higher.

## 6. Therapeutic Management

During the diagnosis of diarrhoea in children, clinical features should be documented to exclude the presence of blood or mucus in the stools. If these are present, then antibiotherapy adapted to the causative pathogen may be required. However, in the case of acute infectious diarrhoea, systematic use of antimicrobial therapy is not recommended because the aetiology may not be bacterial, because the disease is generally self-limiting and due to the risk of development of antibiotic resistance. In such cases, no laboratory tests are necessary to identify the pathogenic agent and a symptomatic treatment can be prescribed straight away. Only if diarrhoea persists despite appropriate symptomatic treatment should patients be evaluated further.

The WHO has set the following therapeutic goals for the treatment of acute diarrhoea [19]:

1. to prevent dehydration,
2. to treat dehydration,
3. to prevent nutritional damage,
4. to reduce the duration and severity of diarrhoea and the occurrence of future episodes.

The mainstay of symptomatic treatment of acute infectious diarrhoea, particularly in young children at risk for dehydration, is to provide rehydration and to continue feeding.

In cases of advanced dehydration, and if needed, fluids can be administered by the intravenous route. The WHO guidelines for treatment of acute diarrhoea in children recommend immediate rehydration comprising corrective electrolyte therapy, maintenance of breastfeeding, or early feeding during a diarrhoea episode [19]. Rehydration can be achieved by oral rehydration solutions (ORS), a mixture of glucose and electrolytes dissolved in water. The composition of ORS has been fixed by the WHO and UNICEF, although this definition has evolved over time. The currently recommended composition is presented in Table 1. Rehydration with ORS is usually sufficient for management of moderate dehydration from acute diarrhoea, regardless of aetiology, which can be safely and effectively treated in over 90% of cases by the use of ORS [19]. ORS is absorbed in the small intestine even during copious diarrhoea, thus replacing the water and electrolytes lost in the faeces. A particular advantage of this is that ORS may be used as home treatment to prevent dehydration [19]. Similarly, early refeeding has been shown to reduce the risk of life-threatening complications of acute watery diarrhoea in children [23].

Nonetheless, even though the use of ORS has drastically reduced mortality rates, it remains underused, with only 33% of children with diarrhoea in developing countries receive ORS to treat their disease [36].

Although it effectively mitigates dehydration, ORS has no effect on the frequency of bowel movements, the duration of diarrhoea, or associated symptoms such as abdominal pain [37]. In this context, adjuvant therapy to rehydration such as micronutrient supplementation (zinc), probiotics, or antidiarrhoeal agents such as antisecretory agents or adsorbent agents may offer a safe complement to ORS in acute mild to moderate infectious diarrhoea to reduce the duration and severity of the symptoms [38, 39]. In 2004, WHO and UNICEF recommended the use of low-osmolarity oral rehydration salts (ORS) in association with zinc tablets to treat all types of diarrhoea among all age groups [2, 40]. The rationale for this was that zinc supplementation may increase the uptake of ORS and reduce the severity and duration of the diarrhoea episode. However, robust data showing the incremental value of zinc salts over ORS alone are scarce and the available data come principally from studies performed in malnourished children in developing countries.

In 2008, the European Society for Paediatric Gastroenterology, Hepatology and Nutrition (ESPGHAN) and the European Society for Paediatric Infectious Diseases (ESPID) joint working group on acute diarrhoea published evidence-based guidelines on the use of antidiarrhoeal drugs to be used as adjunctive therapy to ORS in children [41]. These recommendations emphasised that since it is likely that such drugs will be used in the home setting with little or no medical supervision, candidates should meet the following specifications: (i) be safe and well tolerated, (ii) be usable in conjunction with ORS, (iii) and be effective regardless of the cause of diarrhoea. Four groups of antidiarrhoeal drugs fulfilling these criteria were identified, namely antimotility agents (loperamide), antisecretory agents (racecadotril), probiotics, and adsorbents (diosmectite).

### 6.1. Loperamide

**Figure 2:**
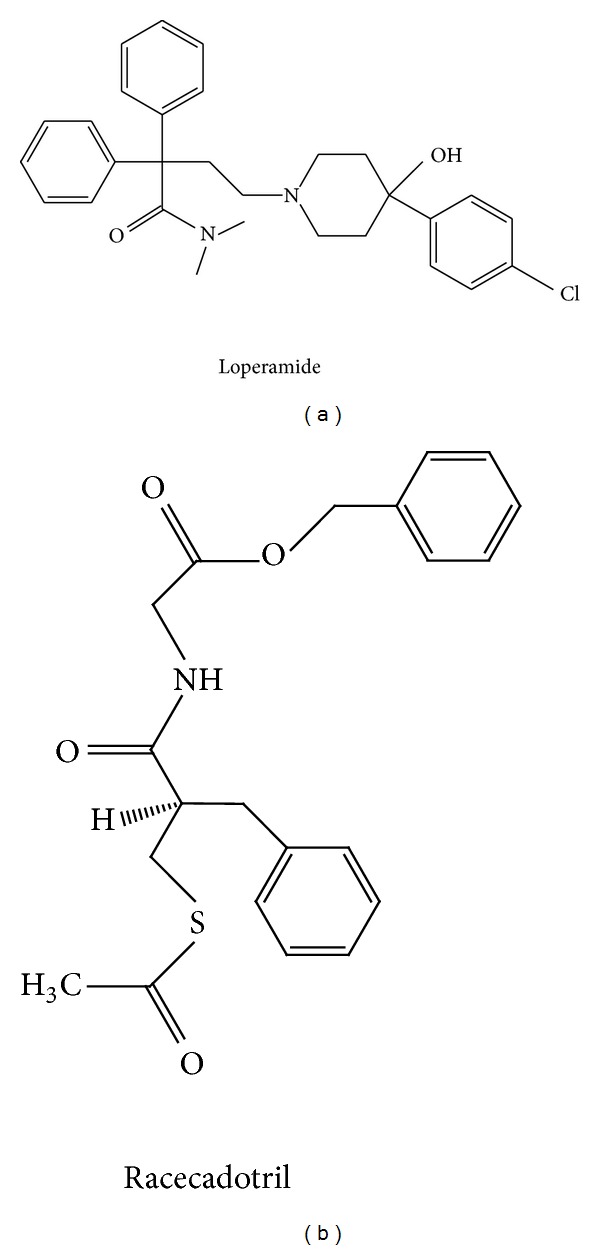
Chemical structures of loperamide and racecadotril.

Loperamide is indicated for the symptomatic treatment of acute diarrhoea in adults and children over 12 years of age and for the symptomatic treatment of chronic diarrhoea in adults [42]. Loperamide is a synthetic opiate agonist (Figure 2) activating the *μ* receptors in the myenteric plexus of the large intestine. These receptors are situated presynaptically on the endings of the parasympathetic cholinergic innervation of the intestinal smooth muscle which exerts a facilitatory effect on smooth muscle contractility [43]. Activation of *μ* receptors by loperamide inhibits release of acetylcholine and thus relaxes smooth muscular tone in the gut wall [44]. The physiological consequence of this is to enhance phasic colonic segmentation and inhibit peristalsis, thus increasing intestinal transit time [42, 45]. In addition, muscarinic acetylcholine receptors on secretory epithelial cells in the gut wall mediate stimulation of secretion of water and electrolytes into the intestinal lumen by parasympathetic activity. Inhibition by loperamide of acetylcholine release will thus also have an antisecretory activity [43]. As a result, loperamide reduces daily faecal volume, decreases fluid and electrolyte loss, and increases stool viscosity and bulk density.

Contrary to the majority of other opiate derivatives, loperamide does not penetrate well into the central nervous system and, for this reason, does not present the sedative side effects and risk of dependence observed with other members of this class [44].

Loperamide is administrated by the oral route (oral solution or capsule) and is rapidly absorbed. Its onset of action is about one hour with a maximum effect at 16–24 h after administration. Absorbed from the gastrointestinal tract, its time to peak plasma concentration is 2.5 hours for the oral solution and 5 hours for capsules. Excreted in the urine (1-2%) and faeces (25–30% as unchanged drug), its elimination half-life is 11 hours [46, 47]. Loperamide is metabolised by the cytochrome P450 (CYP) system and is a substrate for the CYP3A4 isoenzyme.

**Figure 3:**
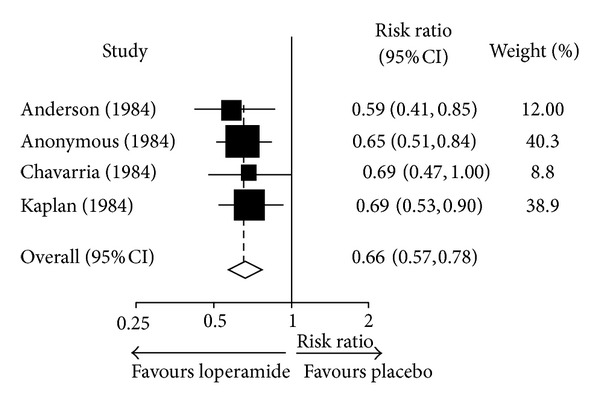
Efficacy of loperamide: meta-analysis of persistence of diarrhoea at 24 h in placebo-controlled clinical trials of loperamide in the treatment of acute diarrhoea in children. The *x*-axis uses the log scale. Risk ratios were calculated using a random-effects model. Reproduced with permission from Li et al. 2007 [48].

A number of clinical trials of varying quality have investigated the potential antidiarrhoeal effects of loperamide. A meta-analysis performed in 2007 evaluated the information available up to that time addressing the efficacy of loperamide in the treatment of acute diarrhoea in children younger than 12 years [48]. This meta-analysis included thirteen randomised controlled trials performed in 1788 patients, but most of these trials had major methodological limitations. The combined data from four of the highest quality of these studies showed that loperamide significantly reduced, compared to placebo, the risk of persistence of diarrhoea at 24 hours (RR: 0.66 (95%CI: 0.57–0.78)) and at 48 hours (RR: 0.59 (0.45–0.78)) (Figure 3). Loperamide also reduced significantly the duration of diarrhoea and the number of stools at 24 hours.

In the trials considered in this meta-analysis, serious adverse events, defined as ileus, lethargy, or death, were reported in 8 out of 972 children allocated to loperamide (0.9% (0.4%–1.7%)) compared with none of 764 children allocated to placebo (0% (0%–0.5%)). These serious adverse events were reported only in children aged under three years. The authors of this meta-analysis concluded that in children under three years, malnourished, moderately or severely dehydrated, systemically ill, or having bloody diarrhoea, adverse events outweigh benefits, while in children older than three years with minimal dehydration, loperamide may be a useful adjunct to oral rehydration and early refeeding [39, 48]. In addition, there have been a number of case reports of paralytic ileus in children treated with loperamide, again usually in patients under three years of age.

Although loperamide is widely used in adult patients and has shown some efficacy in paediatric studies, its use in children has been discouraged by the WHO and the American Academy of Pediatrics due to concerns over its efficacy and safety in young children. The practice guidelines produced by these organisations state that loperamide should not be used in children under twelve years of age [39, 48]. The drug is not approved for use in children in most countries, although in the United States, loperamide is approved by the FDA for use in children above the age of two. Loperamide should never be given if an inflammatory disease is suspected (visible blood in stools, dysentery, or acute colitis).

### 6.2. Racecadotril

Racecadotril (Figure 2) is indicated for the symptomatic treatment of acute diarrhoea in both adults and children. Racecadotril is a prodrug that is rapidly absorbed from the gut and hydrolysed in the plasma to its active metabolite thiorphan [49]. Like loperamide, racecadotril interacts with the opioid neurotransmitter system in the gut wall. Unlike loperamide, this drug does not act at the level of the opiate receptor but rather as an inhibitor of the enzyme neutral endopeptidase 24.11, which is responsible for the degradation of the endogenous opioid peptides Met- and Leu-enkephalin [50, 51]. These enkephalins are endogenous opioid neurotransmitters synthesised and secreted by interneurons of the enteric nervous system, which act on cholinergic neurones, enterochromaffin cells, and secretory epithelial cells to coordinate gastrointestinal function. Whereas loperamide activates principally the *μ* subtype of opiate receptor, enkephalins interact preferentially with the *δ* opiate receptors that are found in high density on secretory epithelial cells. Activation of these receptors leads to reduced secretion of water and electrolytes mediated by a decrease in cellular cAMP [49, 52]. By inhibiting the breakdown of enkephalins, thiorphan facilitates this antisecretory activity. Whereas the antidiarrhoeal effect of loperamide is primarily mediated by reducing gastrointestinal motility through an action at *μ* receptors, that of racecadotril is essentially due to an antisecretory effect mediated by *δ* receptors.

Racecadotril is administrated by the oral route and is rapidly absorbed and converted to thiorphan in the plasma. Maximum plasma thiorphan concentrations are achieved around one hour after administration of racecadotril [53]. Thiorphan does not cross the blood-brain barrier and, for this reason, orally administered racecadotril presents minimal centrally mediated opiate-like effects [49, 53]. Thiorphan is metabolised to inactive metabolites eliminated in the urine.

**Figure 4:**
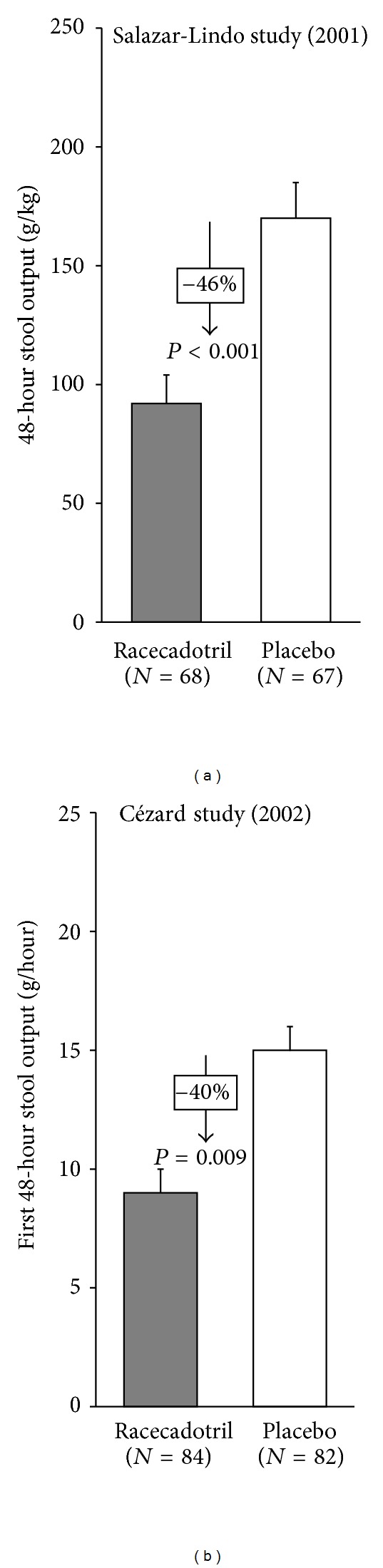
Efficacy of racecadotril at reducing stool output in two randomised placebo-controlled trials.

In clinical trials, racecadotril has been evaluated for the treatment of acute watery diarrhoea in children in a number of trials. The first of these was a large randomised, double-blind study comparing racecadotril to placebo as an adjunctive treatment with ORS in boys under the age of three presenting with acute watery diarrhoea performed in South America [54]. The effectiveness and safety of racecadotril used as an adjunct to ORS for treating acute gastroenteritis in children were reviewed through the meta-analysis conducted by Szajewska et al. in 2007 [55]. The principal outcome measure was stool output, which is considered the most objective and relevant outcome measure for clinical trials in acute diarrhoea [39, 56]. Stool output was reduced by around fifty percent in the racecadotril treatment group (Figure 4). A subsequent study performed in France using a very similar design also reported a similar reduction in stool output [57]. A number of other studies using less rigorous end points and less robust methodologies have generally reported benefits of racecadotril compared to placebo.

**Figure 5:**
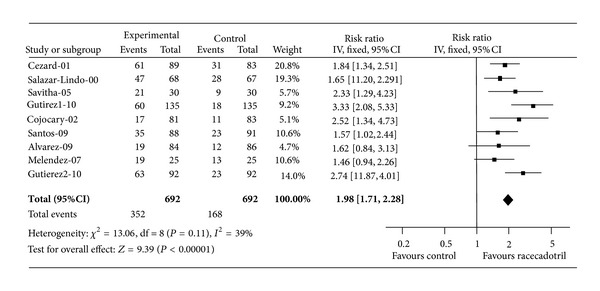
Efficacy of racecadotril: responder rate defined as patients with a short diarrhoea duration (less than 2 days). Summary means adjusted for baseline conditions (rotavirus serostatus, dehydration). Reproduced with permission from Lehert et al. 2011 [58].

In a recent meta-analysis [58], individual patient data from 1384 patients included in nine randomised clinical trials was analysed to compare the efficacy of racecadotril to that of placebo as adjunctive treatment to ORS. The analysis demonstrated clinically relevant benefits of racecadotril with respect to reducing diarrhoea duration (Figure 5), stool output (only two studies evaluated this outcome), and stool number. This meta-analysis identified rotavirus status and baseline dehydration level as important modulators of the treatment response, but found that the observed efficacy of racecadotril was independent of these modulators.

In terms of safety, no adverse events specifically associated with racecadotril have been identified in the published clinical trials. The nature and frequency of adverse events in patients treated with racecadotril were similar to those observed in the placebo group and the frequency of constipation was lower than that observed with loperamide [53]. In particular, there was no evidence for the occurrence of respiratory depression, an adverse event frequently associated with drugs targeting the opioid system.

Finally, a recent cost-utility analysis performed in the UK from the payer perspective has shown that racecadotril used as adjuvant therapy is more effective and less costly compared to ORS alone for the treatment of children with acute diarrhoea [59].

### 6.3. Probiotics

Probiotics have been defined by the joint FAO/WHO Working Group (Food and Agriculture Organisation/World Health Organisation) as “live microorganisms that when administered in adequate amount confer a health benefit on the host” [60]. The mechanism of action of these probiotics is thought to be related to competition in the gut with pathogenic microorganisms for nutrients or adhesion sites, and possibly to secretion by probiotics of molecules that inhibit the growth of pathogenic microorganisms. Probiotic microorganisms are generally administered as spores that can resist transit through the highly acidic *milieu* of the stomach and then germinate and proliferate in the less hostile environment of the intestinal lumen. However, these microorganisms fail to colonise the gastrointestinal tract persistently and they disappear from the faeces within days when supplementation ceases.

**Figure 6:**
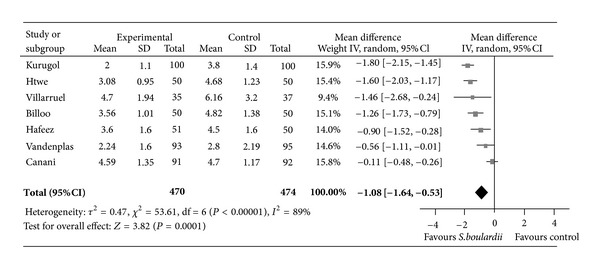
Efficacy of probiotics: weighted mean difference in the duration of diarrhoea between *S. boulardii* and control groups (days). Negative values indicate that the duration of diarrhoea was shorter in the *S. boulardii* group than in the control group. Reproduced with permission from Szajewska and Skórka 2009 [68].

**Figure 7:**
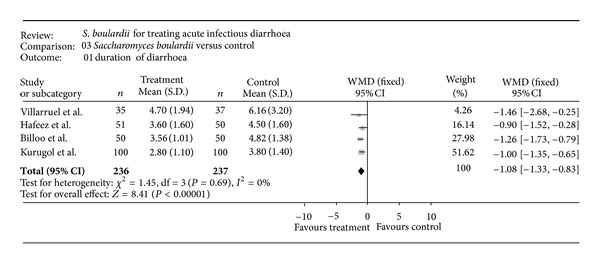
Efficacy of probiotics: mean duration of diarrhoea (hours) with *Saccharomyces boulardii* versus control [69].

The main therapeutic applications of probiotics have been the treatment and prevention of antibiotic-associated diarrhoea. However, there is no evidence of efficacy for most probiotics, and any benefits that have been observed are strain specific and dose dependent [61]. Some meta-analyses have attempted to evaluate the effect of probiotics in the treatment of acute infectious diarrhoea [62–67]. These have concluded that only two probiotic strains presented efficacy in the treatment of acute diarrhoea in children which was statistically significant effect and of moderate clinical benefit. In particular, *Saccharomyces boulardii* [63] and *Lactobacillus *GG [63] have shown efficacy on the duration of childhood diarrhoea and on stool consistency (Figures 6 and 7) but not on the more robust criterion of stool output.

Although major safety issues are not generally associated with probiotics, there is a potential risk of septicaemia or deep tissue infection if these microorganisms escape from the “protected” environment of the gut and disseminate through the organism [71]. There is also a theoretical risk that probiotics may release toxins that are detrimental to the host. In addition, there is evidence of the development of antibiotic resistance for some probiotic strains, notably *Lacobacillus reuteri* and *Enterococcus faecium*, and the transfer of this resistance to the native gut flora of the intestinal tract [72–74].

### 6.4. Diosmectite

Diosmectite is an adsorbent clay mineral indicated for the treatment of acute and chronic diarrhoea. It is a natural multilamellar clay, belonging to the dioctahedral smectite class and consists of a double aluminium and magnesium silicate arranged in parallel leaflets [75, 76]. Diosmectite is administrated by the oral route and is not absorbed following its ingestion but acts in the intestinal lumen. Diosmectite is eliminated unchanged directly through the faeces within sixteen hours of administration.

Diosmectite has been demonstrated to have several pharmacological properties which may be beneficial for the treatment of acute diarrhoea [77]. Firstly, diosmectite can adsorb bacterial toxins, bacteria, viruses, bile salts, and lysolecithins. These include enteropathogens such as *E. coli*, rotavirus, and coronavirus as well as bacterial toxins such as *Clostridium difficile* enterotoxins A, B, and C and *C. perfringens* enterotoxin [78]. One study in healthy volunteers demonstrated that diosmectite could reduce the production of hydrogen in the colon during microbial fermentation [79]. Secondly, due to its multilayer structure and its high plastic viscosity, diosmectite possesses powerful coating properties. The preserved integrity of the mucus layer can render the intestinal epithelium more resistant to attack by endogenous (such as pepsin and bile salts) or exogenous (such as bacterial toxins, NSAIDs, and alcohol). This has been demonstrated in animal models [77] and in humans [80]. Thirdly, diosmectite may affect intestinal permeability and electrolyte flux, perhaps as a consequence of its protective effect on the gastrointestinal epithelium [77]. This has been observed in children with acute diarrhoea as determined in by the lactulose-mannitol intestinal permeability challenge test [81].

Finally, diosmectite appears to have a protective effect against intestinal inflammation. Diosmectite has been demonstrated to suppress the production of cytokines such as interleukin-8 from secretory epithelial cells [82] in vitro and to attenuate the proinflammatory action of TNF*α* [83].

**Figure 8:**
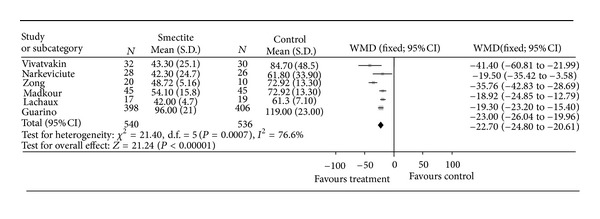
Mean duration of diarrhoea (h) in comparative randomised clinical studies of diosmectite. Reproduced from Szajewska et al. 2006 [70] with permission.

Over a dozen studies performed with diosmectite in children and infants have provided evidence for the efficacy of this medication in the treatment of acute diarrhoea, although these studies have been of variable quality. In a systematic review published in 2006 [70], Szajewska et al. evaluated nine randomised controlled trials including 1238 participants by meta-analysis. All these studies were carried out in children under five years and the majority in infants under two years. The main criterion of the meta-analysis was the duration of diarrhoea. This could be assessed in six of the included studies, which enrolled between them 1076 patients. The findings of all these six studies concurred and demonstrated a significant reduction in the duration of diarrhoea in patients treated with diosmectite (Figure 8). The standard mean difference in diarrhoea duration was 22.7 hours (95% CI: −24.8 to −20.6) in favour of the diosmectite + ORS group, compared with the ORS alone group. This difference was both statistically significant (*P* < 0.0001) and clinically relevant. The meta-analysis also evaluated the proportion of patients recovered after three and five days of treatment, which could be evaluated from four studies. Again, all studies were consistent, showing a higher recovery rate in patients treated with diosmectite. Using a random-effects model, the relative chance of recovery was 1.55 (95% CI: 1.29–1.87) on Day 3 and 1.19; (95% CI: 0.93–1.53) on Day 5. This corresponds to a numbers needed to treat on Day 3 of 4, which can be considered as favourable. The lack of a significant treatment effect on Day 5 can probably be accounted for by the natural course of infectious diarrhoea, which usually resolves spontaneously over this time frame.

Since the publication of this meta-analysis, two large randomised placebo-controlled trials have evaluated diosmectite as an adjunctive therapy to ORS, with one conducted in Peru and the other in Malaysia [84]. These two studies used the same primary outcome measure (72-hour stool output). Between them, the trials included 602 boys aged up to 36 months. In both studies, stool output was significantly reduced in the diosmectite group compared to the placebo group.

In these two studies, patients were stratified by rotavirus infection status. A greater cumulative stool output was observed in the rotavirus-positive subgroup than in the rotavirus-negative subgroup, as well as a larger difference in stool output between the diosmectite and placebo treatment groups (Table 2). In a secondary analysis of date pooled from the two studies, an analysis of variance with three factors (rotavirus status, study, and treatment) on the primary efficacy outcome variable identified significant effect of rotavirus status (*P* < 0.0001) and treatment (*P* = 0.0016) and a significant interaction between treatment and rotavirus status (*P* = 0.0011). Diarrhoea duration was a secondary efficacy criterion. In both studies, the time to recovery was significantly shorter (*P* ≤ 0.01; logrank test) in the diosmectite treatment group compared to the placebo treatment group (68.2 hours versus 118.9 hours in the Peru study and 23.8 versus 31.0 hours in the Malaysia study). This difference of two days (43% reduction) in the former study and of seven hours (23% reduction) in the latter represents an important and clinically relevant reduction in diarrhoea duration.

Concerning safety, adverse events were documented systematically in the two recent randomised trials comparing diosmectite to placebo [84]. No differences in the nature or frequency of adverse events between the diosmectite and placebo group were observed. This is consistent with safety data reported from previous studies [70]. The only adverse event that could be imputed to diosmectite was the emergence or worsening of constipation.

## 7. Treatment Guidelines

Practice guidelines for the treatment of acute diarrhoea in children have been issued by a number of healthcare organisations. As indicated above, the 2005 WHO guidelines [19] focus on the use of ORS as the cornerstone of management of uncomplicated acute diarrhoea in children, together with zinc supplementation. These guidelines consider that antidiarrhoeal drugs have no practical benefit in the treatment of this condition. More recently, the WHO has recommended systematic vaccination against rotavirus in order to reduce the incidence of acute infectious diarrhoea in children [85], following the demonstration that rotavirus immunisation can be highly protective against severe gastroenteritis episodes in developing and developed countries and can also decrease the severity of diarrhoea leading to less death and hospitalization [86–90]. However, anti-rotavirus immunisation does not diminish the need to treat diarrhoeal episodes when they do occur, regardless of cause, nor protect against gastroenteritis caused by other pathogens.

The 2008 practice guideline of the World Gastroenterology Organisation (WGO) [1] follows the WHO guideline in considering that antidiarrhoeal drugs are of little practical benefit in children with acute diarrhoea. It specifies that loperamide is not recommended for children under two years of age and that racecadotril and bismuth salicylate can reduce stool output in children with diarrhoea and may thus be useful. For diosmectite, the WGO considers that the proof of efficacy is inadequate, but it should be noted that these guidelines predate the two large randomised trials that evaluated this agent on stool output. Concerning probiotics, the guidelines considered that available data support the use of specific probiotic strains in the treatment and prevention of rotavirus diarrhoea in infants, but caution against extrapolating results between probiotic strains.

The European Society for Paediatric Gastroenterology, Hepatology and Nutrition (ESPGHAN) and the European Society for Paediatric Infectious Diseases (ESPID) published evidence-based guidelines for the management of acute gastroenteritis in children in Europe in 2008 [41]. In contrast to the WHO and WGO guidelines, these guidelines do evaluate pharmacological antidiarrhoeal treatments, although they state that such drugs are generally not necessary. The recommendations relating to the antidiarrhoeal agents evaluated in the present paper are reproduced in Table 3. The grade of recommendation was considered to be B, being supported by Level II evidence which requires strong evidence from at least one properly designed randomised controlled clinical trial of appropriate size.

## 8. Conclusion

Acute diarrhoea in children is a major public health burden associated with considerable health costs, both for families and public health organisations and programs. Despite a marked improvement in the accessibility of health services, diarrhoeal illness still causes deaths in children, mainly in developing countries. It also has long-term consequences on growth and on physical and cognitive development. Correction of dehydration with ORS and maintenance of good nutritional status are the primary goals of treatment. Adjuvant use of drugs whose safety and effectiveness have been well evaluated and clearly demonstrated can be of use in reducing the severity and duration of diarrhoeal episodes but not hospitalization rates and mortality. Appropriate management of acute diarrhoea with adequately validated treatments may help reduce the health and economic burden of acute diarrhoea in children worldwide.

## Figures and Tables

**Table 1:** Composition of oral rehydration salt solutions as defined in the 2009 WHO/UNICEF recommendations.

Glucose	75 mmol/L
Sodium	75 mmol/L
Chloride	65 mmol/L
Potassium	20 mmol/L
Citrate	10 mmol/L
Osmolarity	245 mmol/L

**Table 2:** Stool output measured in two randomised, placebo-controlled studies of diosmectite for the treatment of acute diarrhoea in children. Data are presented in g/Kg body weight and according to rotavirus status.

	Peru study			Malaysia study		
	Placebo	Diosmectite	*P*	Placebo	Diosmectite	*P*
Rotavirus positive	188 ± 122	147 ± 90	0.0386	185 ± 192	92 ± 103	0.0016
Rotavirus negative	90 ± 52	98 ± 69	0.4884	79 ± 66	87 ± 78	0.4338
All patients	119 ± 93	102 ± 66	0.0315	91 ± 94	88 ± 81	0.0071

**Table 3:** Overview of benefits and risks of anti-diarrhoeal drugs based on ESPGHAN/ESPID guidelines [39].

Efficacy	Safety	Recommendations
*Loperamide *		
Combined data from four RCTs showed that loperamide compared with placebo reduced the risk of (i) diarrhoea at 24 hours and at 48 hours. (ii) Loperamide also reduced the duration of diarrhoea (6 trials), and the number of stools at 24 hours (4 trials).	(i) In the considered studies, serious adverse events, defined as lethargy or death, were reported in 8 out of 972 children allocated to loperamide compared with none of 764 children allocated to placebo. (ii) All serious adverse events were reported in children less than 3 years of age. (iii) Loperamide may exert life-threatening effects and, for this reason, it should not be used for the management of acute diarrhoea in infants and young children.	Loperamide should not be used in the management of acute diarrhoea in children.
*Racecadotril *		
(i) In three relatively small RCTs with some methodological problems, two conducted in hospitalised children, in developed and developing countries, racecadotril was effective in reducing the volume and frequency of stool output and in reducing the duration of diarrhoea (particularly in children with rotavirus diarrhoea). (ii) There is evidence in favour of the use of racecadotril over placebo or no intervention to reduce the stool output in children with acute diarrhoea.	(i) Tolerability of racecadotril was good in these studies. (ii) Racecadotril did not differ from placebo in terms of adverse events, none of which was severe. (iii) The available evidence base does not take into account safety concerns that can be resolved either in studies involving large cohorts of children or in post-marketing surveillance evaluation, which is mandatory before therapy with racecadotril can be recommended.	(i) May be considered in the management of acute diarrhoea in children. (ii) However, well-designed prospective studies of efficacy and safety should be carried out in outpatient children.
*Diosmectite *		
(i) The results of one meta-analysis are promising, and the use of diosmectite may be considered in the management of acute diarrhoea as an adjunct to standard rehydration therapy.		
(ii) These results should be interpreted with caution, because most of the included studies had important limitations.	*Not discussed. *	Diosmectite may be considered in the management of acute diarrhoea in children.
(iii) Cost-effectiveness analyses should be undertaken before routine pharmacological therapy with diosmectite is universally recommended.		
(iv) It is important to delineate the groups (out-patient versus in-patient, older versus younger, viral versus other aetiology of diarrhoea) that derive the greatest clinical benefit from diosmectite therapy.		
*Probiotics *		
(i) Data from several meta-analyses consistently show a statistically significant effect and moderate clinical benefit of selected probiotic strains in the treatment of acute watery diarrhoea (primarily rotaviral), mainly in infants and young children.	(i) Safety issues with probiotics are related to bacterial translocation and sepsis and to the risk of antibiotic resistance.	(i) Probiotics may be an effective adjunct to the management of diarrhoea. However, because there is no evidence of efficacy for many preparations, we suggest the use of probiotic strains with *proven efficacy* and in appropriate doses for the management of children with acute gastroenteritis as an adjunct to rehydration therapy.
(ii) The beneficial effects of probiotics in acute diarrhoea in children seem to be moderate, strain-dependent and dose-dependent.	(ii) While bacterial translocation seems an exceptional event, antibiotic resistance may be a true problem in terms of safety.	(ii) The following probiotics showed benefit in meta-analyses of RCTs: *Lactobacillus *GG and *Saccharomyces boulardii. *
		(iii) Evidence of lack of risk of antibiotic resistance transfer is required for probiotics proposed for clinical use.
